# Solanine Inhibits Proliferation and Angiogenesis and Induces Apoptosis through Modulation of EGFR Signaling in KB-ChR-8-5 Multidrug-Resistant Oral Cancer Cells

**DOI:** 10.3390/jcm13154493

**Published:** 2024-07-31

**Authors:** Prathibha Prasad, Mohamed Jaber, Tahani Awad Alahmadi, Hesham S. Almoallim, Arun Kumar Ramu

**Affiliations:** 1 Medical and Dental Sciences Department, College of Dentistry, Center of Medical and Bio-Allied Health Sciences Research, Ajman University, Ajman P.O. Box 346, United Arab Emirates; 2 Department of Oral Pathology, Saveetha Dental College and Hospital, Saveetha Institute of Medical and Technical Sciences (SIMATS), Saveetha University, Chennai 600077, India; 3 Clinical Dental Sciences, College of Dentistry, Center of Medical and Bio-Allied Health Sciences Research, Ajman University, Ajman P.O. Box 346, United Arab Emirates; mohamed.jaber@ajman.ac.ae; 4 Department of Pediatrics, College of Medicine and King Khalid University Hospital, King Saud University, Medical City, P.O. Box 2925, Riyadh 11461, Saudi Arabia; talahmadi@ksu.edu.sa; 5 Department of Oral and Maxillofacial Surgery, College of Dentistry, King Saud University, Riyadh 11545, Saudi Arabia; hkhalil@ksu.edu.sa; 6 Department of Biochemistry and Biotechnology, Centre for Research and Development Ponnaiyah Ramajayam Institute of Science and Technology (PRIST Deemed University), Thanjavur 613403, India

**Keywords:** solanine, multidrug resistance, oral cancer, EGFR signaling, apoptosis

## Abstract

**Background:** The most important factors contributing to multi-drug resistance in oral cancer include overexpression of the EGFR protein and the downstream malignancy regulators that are associated with it. This study investigates the impact of solanine on inflammation, proliferation, and angiogenesis inhibition in multidrug-resistant oral cancer KB-Chr-8-5 cells through inhibition of the EGFR/PI3K/Akt/NF-κB signaling pathway. **Methods:** Cell viability was assessed using an MTT assay to evaluate cytotoxic effects. Production of reactive oxygen species (ROS), mitochondrial membrane potential (ΔΨM), and AO/EtBr staining were analyzed to assess apoptosis and mitochondrial dysfunction. Western blotting was employed to examine protein expression related to angiogenesis, apoptosis, and signaling pathways. Experiments were conducted in triplicate. **Results:** Solanine treatment at concentrations of 10, 20, and 30 μM significantly increased ROS production, which is indicative of its antioxidant properties. This increase was associated with decreased mitochondrial membrane potential (ΔΨM) with *p* < 0.05, suggesting mitochondrial dysfunction. Inhibition of EGFR led to reduced activity of PI3K, Akt, and NF-κB, resulting in decreased expression of iNOS, IL-6, Cyclin D1, PCNA, VEGF, Mcl-1, and HIF-1α and increased levels of the apoptotic proteins Bax, caspase-9, and caspase-3. These changes collectively inhibited the growth of multidrug-resistant (MDR) cancer cells. **Conclusions:** Solanine acts as a potent disruptor of cellular processes by inhibiting the EGFR-mediated PI3K/Akt/NF-κB signaling pathway. These results suggest that solanine holds promise as a potential preventive or therapeutic agent against multidrug-resistant cancers.

## 1. Introduction

Multidrug resistance (MDR) poses a significant challenge in oral cancer therapy, contributing to tumor recurrence, angiogenesis, and metastasis [1]. Over 90% of deaths among cancer patients receiving traditional chemotherapeutics or novel targeted drugs are attributed to multidrug resistance [2]. Tobacco use, inadequate diet, and genetic predispositions contribute to the development of drug-resistance factors, underscoring their importance in the progression of oral squamous cell carcinoma (OSCC) [3,4]. It has been nearly four decades since the five-year survival rate for OSCC dropped below 50% despite the use of several therapeutic approaches, individually and in combination [5]. In order for this hallmark to progress, various intracellular signaling pathways must be disrupted. In particular, oncogene receptor modulation and altering crosstalk with cancer regulators are critical and difficult tasks in cancer chemotherapy.

The overexpression of the oncogenic epidermal growth factor receptor (EGFR) has been implicated in oral squamous cell carcinomas as playing a pivotal role in driving tumor progression, including aspects such as cell proliferation, metastasis, and resistance to chemotherapy [6]. The activated EGFR protein acts as a catalyst for numerous downstream signaling pathways, notably the PI3K/AKT/NF-κB pathways, which are recognized as critical components in this signaling cascade. Inflammatory, proliferative, and angiogenic responses have been associated with the activation of these pathways in combination, promoting tumor progression and drug resistance [7]. Therefore, in order to develop therapeutic approaches against the development of drug resistance in OSCC, it is crucial to target dysregulated EGFR and its associated regulators.

Recent investigations have unveiled the promise of chemicals targeting dysregulated EGFR and its regulators as an alternative approach to combating cancer, a potential primarily attributed to their capacity to thwart multiple signaling pathways [8]. Emerging research underscores the remarkable ability of various phytochemicals derived from medicinal plants to reduce cellular proliferation, inhibit angiogenesis and metastasis, and induce apoptosis in drug-resistant cancer cells [9]. Solanine, a steroidal alkaloid naturally found in the Chinese herb *Solanum nigrum* L., has been demonstrated to possess significant anticancer properties, making it widely utilized as an anticancer compound in clinical practice [10]. Solanine exhibits antioxidant, anti-inflammatory, and antimicrobial properties [11,12]. According to a recent report, solanine appears to inhibit the growth of adipogenesis-associated Mth938 domain-containing (AAMDC) gastric cancer cells without having an adverse effect on normal cells [13]. Zheng et al. 2020 revealed that cell proliferation is inhibited and apoptosis promoted by treatment with solanine through suppression of miR-16/Bcl-2 signaling in human leukemia cells [14]. Several strains of evidence suggest that solanine may be a new and effective cancer treatment or adjuvant therapy, even if there is a lack of large-scale clinical trials on it to date [15]. However, solanine’s ability to suppress drug-resistant cancer cells through its interactions with extracellular and intracellular molecules is not well understood.

This study’s primary goal is to understand how solanine modulates the EGFR/PI3K/Akt/NF-κB signaling pathway in multidrug-resistant oral cancer cells (KB-ChR-8-5). The purpose is to elucidate the mechanisms by which solanine induces apoptosis, inhibits proliferation, and reduces angiogenesis in these resistant cancer cells. We hypothesize that solanine treatment will result in the downregulation of EGFR and its downstream signaling pathways, leading to increased production of reactive oxygen species (ROS), mitochondrial dysfunction, and the induction of apoptosis in KB-ChR-8-5 cells.

## 2. Material and Methods

### 2.1. Reagents and Antibodies

Rhodamine 123, Dulbecco’s Modified Eagle Medium (DMEM), trypsin EDTA, glutamine, Hoechst 33342, ethidium bromide (EtBr), phosphate-buffered saline (PBS), penicillin-streptomycin solution (antibiotics), 2-mercaptoethanol, fetal bovine serum (FBS), 3-(4,5-dimethylthiazol-2-yl)-2,5-diphenyl tetrazolium bromide (MTT), 2,7-diacetyl-dichloro fluorescein (DCFH-DA), and acridine orange (AO) were procured from Hi-media. Solanine (S3757), as well as monoclonal antibodies for cyclin-D1, Akt, PI3k, IκBα, HIF-1α, VEGF, iNOS, EGFR, IL-6, PCNA, and NF-κB and the polyclonal antibody IgG-HRP were sourced from Sigma Chemicals Co., St. Louis, MO, USA.

### 2.2. Culturing Cells

KB-ChR-8-5 cells, which are drug-resistant and which originated from human oral cancer, and normal fibroblast L929 cells were sourced from the National Centre for Cell Sciences (NCCS) in Pune, India. For this study, Dulbecco’s Modified Eagle Medium (DMEM) was employed, supplemented with 100 units per milliliter of penicillin and streptomycin, along with 10% fetal bovine serum. These cells were cultured under standard conditions at 37 °C in an environment maintained with 5% CO_2_ and controlled relative humidity.

### 2.3. Cell-Viability Assessment and Cytotoxicity Assay

To assess cell viability in response to solanine, we employed the colorimetric MTT assay. Initially, cells were seeded in 96-well culture plates at a density of 1 to 10^4^/mL (100 μL each well) after trypsinization. After a 24 h incubation period, the culture medium was aspirated and solanine (0.5% *v*/*v* DMSO) solutions at concentrations ranging from 0 to 100 μM were added to the wells [16]. Following a 24 h treatment period, the adherent cells were exposed to MTT solution (0.5 mg/mL) for 4 h. Subsequently, the culture medium was removed and 100 μL of dimethyl sulfoxide was added to each well. The absorbance of the microplates was measured at a wavelength of 570 nanometers using a Microplate Reader (BIO-RAD, Hercules, CA, USA). All experiments were conducted in triplicate.

### 2.4. Determination of Intracellular ROS Generation

Intracellular reactive oxygen species (ROS) were detected using 2,7-diacetyl dichlorofluorescein (DCFH-DA), a compound capable of penetrating the intracellular matrix. Within the cells, ROS caused the oxidation of DCFH-DA, leading to the formation of a fluorescent compound known as DCF, which plays a pivotal role in this biological process, as indicated by Jesudason et al. [17]. For the experimental setup, drug-resistant OSCC KB-ChR-8-5 cells (1 × 10^6^ per well) were cultivated in six-well plates and allowed to mature over a 24 h period within a controlled CO_2_ incubator. Subsequently, after this 24 h maturation phase, the cells were subjected to solanine (10, 20, and 30 μM) treatment, which was followed by staining with DCFH-DA for a duration of 10 min. This treatment ultimately yielded an increase in fluorescence intensity, which was recorded by Image J Software (V.1.49, NIH, Bethesda, MD, USA) and expressed as a percentage (%). Finally, to quantify the fluorescence intensity, measurements were carried out using a fluorescence microplate reader (Tecan, Infinite M1000, Mannedorf, Switzerland) with either a 485 nm excitation filter or a 535 nm emission wavelength. Using an Olympus fluorescent microscope (Olympus, Tokyo, Japan), cells were observed. All experiments were performed in triplicate.

### 2.5. Monitor the Loss in Mitochondrial Membrane Potential

In this research study, mitochondrial membrane potential (MMP) was assessed using the lipophilic cationic dye, rhodamine-123. KB-ChR-8-5 cells were cultivated in a six-well plate at a seeding density of 1 × 106 cells per well and treated with solanine (10, 20, and 30 μM) for a period of 24 h. Subsequently, the cells were subjected to a 30 min incubation with the fluorescent dye Rh-123 (10 μg/mL) [18]. Following this incubation, the cells were trypsinized and the fluorescence intensity was quantified by a fluorescence microplate reader (Infinite M1000, Tecan) at emission wavelengths of 485/585 nm, respectively. A fluorescent microscope (Olympus, Tokyo, Japan) was used to take the images. All the experiments were performed in triplicate.

### 2.6. Detection of Morphological Changes Linked to Apoptosis

The investigation of apoptotic features through morphological analysis was carried out by employing a staining technique using acridine orange and ethidium bromide (AO/EtBr). Cells (1 × 106) were subjected to a 24 h solanine (10, 20, and 30 μM) treatment and subsequently exposed to a dye mixture composed of acridine orange and ethidium bromide. To complete the staining process, the cells were incubated for 30 min at a temperature of 37 °C [19]. The stained cells were immediately examined using a fluorescent microscope from Olympus, Japan, and the emission wavelengths of AO (450–490 nm) and EtBr (500–700 nm). All experiments were performed in triplicate

### 2.7. Immunoblot Analysis

The cell samples underwent RIPA lysis-buffer treatment for approximately 30 min, which was followed by a 4 °C centrifugation step of the same duration. The supernatant was carefully removed, and the proteins were extracted. Protein concentration was assessed using a nanodrop spectrophotometer (Thermo Scientific, Waltham, MA, USA). Subsequently, each protein sample was mixed with sample SDS buffer and denatured for approximately 5 min. These denatured proteins were then separated through SDS-PAGE electrophoresis and transferred onto a PVDF membrane using a semi-dry blotting method. The PVDF membrane was subsequently subjected to blocking using 5% BSA and was incubated overnight with specific antibodies directed against the proteins of interest at 4 °C. Following this, the membrane underwent rinsing with 1 × TBST and was treated with horseradish peroxidase (HRP)-conjugated secondary antibodies for 1 h at room temperature. It was then washed three times with 1 × TBST and subjected to detection using a chemiluminescent detection system [20]. All the experiments were performed in triplicate.

### 2.8. Statistical Analysis

The statistical analysis was carried out using the ANOVA test. Statistical analysis and graph creation were performed using SPSS version 25 (SPSS Inc., Chicago, IL, USA). Statistical significance was denoted as * *p* < 0.05.

## 3. Result

### 3.1. Evaluation of the Impact of Solanine on Cell Viability

**Figure 1:**
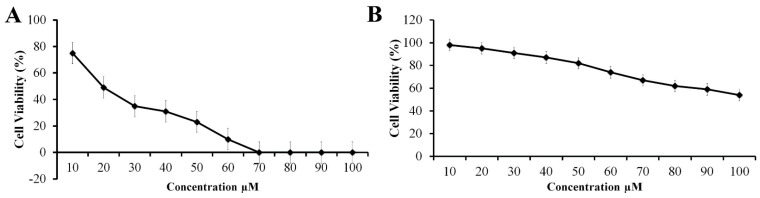
Evaluation of cytotoxic activity of solanine in human (**A**) multidrug-resistant oral cancer KB-ChR-8-5 cells and (**B**) normal fibroblast L929 cells. The MTT test was used to assess the viability of the cells after they had been exposed to various concentrations of solanine for 24 h. All data are presented as mean values ± standard deviations and are representative of at least three independent experiments.

T the effects of solanine at different doses (0 to 100 μM) on KB-ChR-8-5 oral cancer cells and normal fibroblast L929 cells were examined to determine whether the solanine was specifically targeting cancer cells rather than normal cells. The results showed that cancer cells were more sensitive to solanine than were normal cells. Notably, a solanine concentration of 60 μM markedly increased the degree of cell death. Hence, the inhibitory concentration 50 (IC50) of solanine for KB-ChR-8-5 cells was determined to be 30 μM. Based on the growth-inhibition curve, we selected doses of 10, 20, and 30 μM for further investigations. Interestingly, solanine treatment did not significantly impact cell viability in normal fibroblast L929 cells (Figure 1). This differential response underscores solanine’s potential for selectivity towards cancer cells while sparing normal cells, highlighting its promising therapeutic profile.

### 3.2. Impact of Solanine on the Generation of Reactive Oxygen Species (ROS)

**Figure 2:**
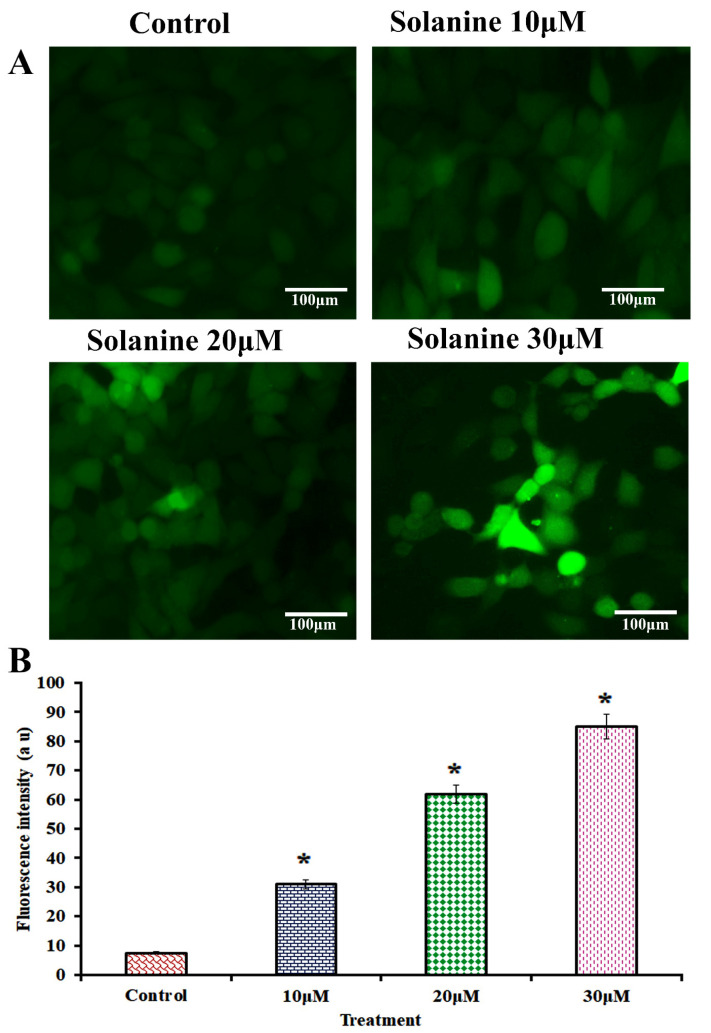
The effect of solanine on the production of intracellular ROS in KB-ChR-8-5 cells was investigated via the DCFH-DA-staining technique. (**A**) On the basis of the micrographs, it was discovered that untreated KB-ChR-8-5 cells had weak DCF fluorescence and that different doses of solanine (10, 20, and 30 µM) resulted in enhanced generation of ROS, suggesting a significant amount of DCF fluorescence intensity. The FLoid Cell Imaging Station captured this picture of cells (40× magnifications, scale bar = 100 µm). (**B**) A spectrofluorometer may be used to determine the amount of ROS production. Except as otherwise stated, all experiments were conducted in triplicate, and the results are represented as the mean standard deviation (mean ± SD). The statistical significance of the results was determined using an analysis of variance. The data are presented as the mean ± SD (*n* = 3). * *p* < 0.05 compared with the control.

The study of solanine’s effect on the production of reactive oxygen species (ROS) is depicted in Figure 2. The concentrations of solanine used, 10, 20, and 30 μM, effectively enhanced ROS generation, as determined by the DCFH-DA dye assay. The results demonstrate that solanine treatment resulted in a significant increase in ROS production in the KB-ChR-8-5 cells, which was confirmed by the emission of prominent fluorescence as compared to control cells. These findings imply that increased ROS generation is responsible for the induction of apoptosis through mitochondrial damage. Therefore, it can be inferred that ROS plays a crucial role in the cell-death process induced by solanine.

### 3.3. Effect of Solanine on Mitochondrial Membrane Potential (MMP)

**Figure 3:**
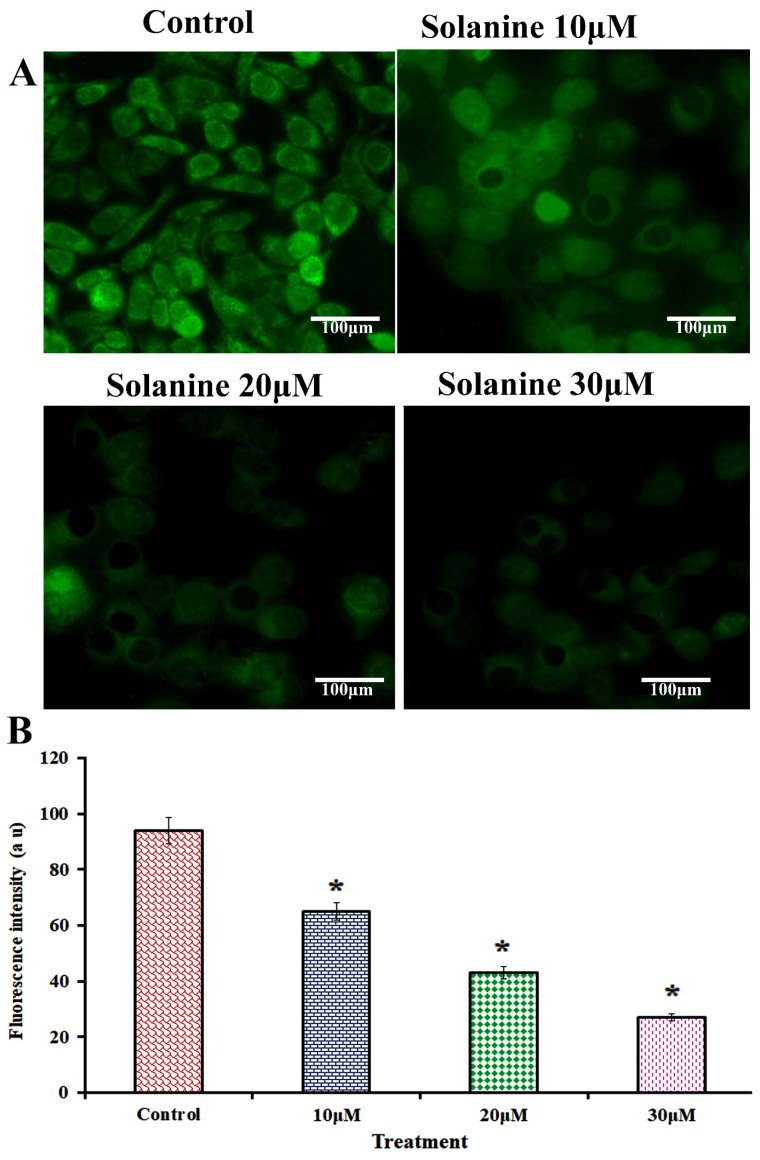
To investigate the effects of solanine on mitochondrial membrane potential (MMP) in KB-ChR-8-5 cells, rhodamine 123 staining was used. (**A**) In the KB-ChR-8-5 untreated cells, strong fluorescence was seen, indicating polarization of the mitochondrial membrane. Different doses of solanine (10, 20, and 30 μM) administered over a 24 h period suggest the collapse of the mitochondrial matrix. A FLoid Cell Imaging Station captured this picture of cells (40× magnification, scale bar = 100 µm). (**B**) The spectrofluorometer was used to determine the intensity of the fluorescence. All experiments were conducted in triplicate, and the results are represented as the mean ± the standard deviation (mean ± SD). The statistical significance of the results was determined using an analysis of variance. The data are presented as the mean ± SD (*n* = 3). * *p* < 0.05 compared with the control.

The effect of solanine on the MMP levels in KB-ChR-8-5 cells is shown in Figure 3. Rhodamine 123 was used to evaluate the mitochondrial membrane after solanine exposure at 10, 20, and 30 μM. The treated cells show a notable decrease in green fluorescence intensity when compared to the control, suggesting that the loss of membrane integrity was dose-dependent and resulted in Cyt-c’s release from the mitochondria.

### 3.4. Effects of Solanine on Apoptotic Morphological Alterations

**Figure 4:**
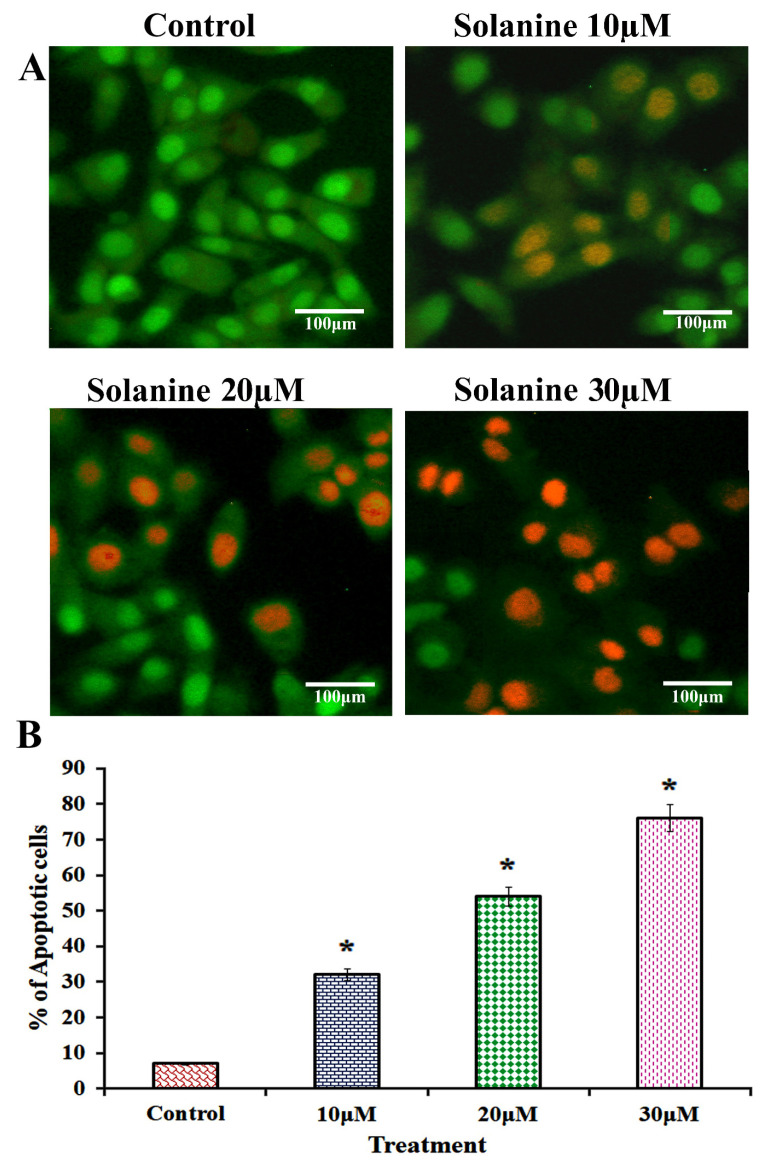
Apoptosis was seen in the presence of solanine, as determined by dual staining (AO/EtBr) in the presence of solanine. (**A**) KB-ChR-8-5 cells were treated with solanine at varied doses (10, 20, and 30 μM), and the percentage of apoptotic cells in each treatment group significantly increased as compared to untreated cells (40× magnifications, scale bar = 100 µm). (**B**) To show that the proportion of apoptotic cells has been calculated, the data are given as the mean ± the standard deviation of three separate experiments (SD). All experiments were conducted in triplicate, and the results are represented as the mean ± the standard deviation (mean ± SD). The statistical significance of the results was determined using an analysis of variance. The data are presented as the mean ± SD (*n* = 3). * *p* < 0.05 compared with the control.

The nuclei were primarily observed to be fragmented into smaller pieces, which is a definite indication that apoptotic bodies had formed in KB-ChR-8-5 cells that had been exposed to solanine. The orange-red stain indicates that exposure to different doses of solanine (10, 20, and 30 μM) resulted in a greater number of late apoptotic cells, indicating damage to both the cell membrane and DNA (Figure 4). These changes were not observed in vehicle control cells, which displayed stained nuclei with an intact cellular structure and green fluorescence. The AO staining method is a reliable means of distinguishing between living and dead cells, while EtBr staining is suitable for identifying dead cells. As a result, our findings suggest that solanine has a significant impact on nuclear morphology, indicative of solanine-induced apoptosis.

### 3.5. Impact of Solanine on the Expression of Proteins Related to Inflammation and Proliferation

**Figure 5:**
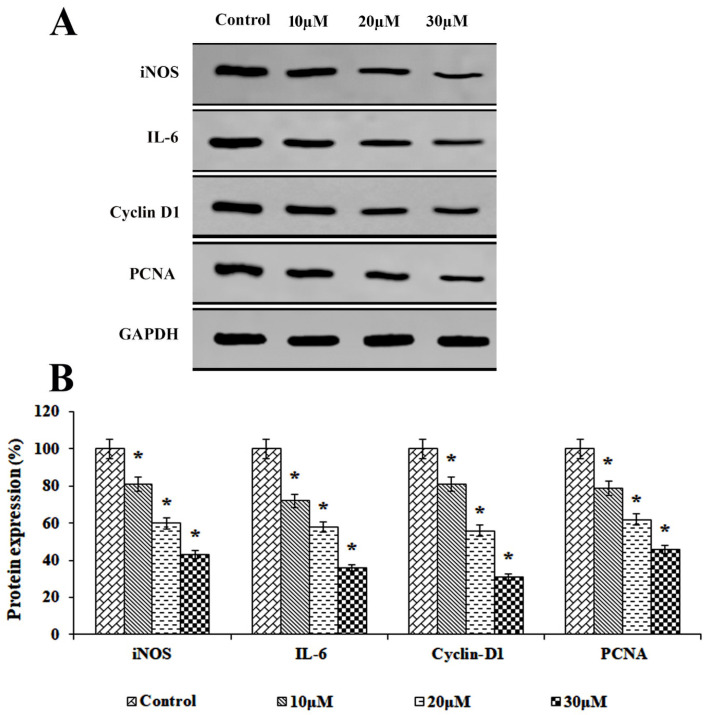
Effects of solanine on the expression of proteins associated with inflammation and proliferation. (**A**) The expression of iNOS, IL-6, cyclin-D1, and PCNA in KB-ChR-8-5 cells after 24 h of treatment with and without solanine is shown in the representative immunoblot study. GAPDH served as a loading-control protein. (**B**) Densitometric analysis. Protein expression in the control lysates was assessed in triplicate and is represented as 100% in the graph. The statistical significance of the results was determined using an analysis of variance. The data are presented as the mean ± SD (*n* = 3). * *p* < 0.05 compared with the control.

The research results have demonstrated that solanine exerts an inhibitory effect on the expression of inflammatory and proliferative proteins, as determined through the Western blot analysis presented in Figure 5. The results indicate that varying solanine concentrations (10, 20, and 30 μM) considerably reduce the expression of iNOS, IL-6, cyclin-D1, and PCNA proteins in KB-ChR-8-5 cells, suggesting that solanine has the ability to significantly decrease the formation of malignant tumors.

### 3.6. Effect of Solanine on the Expression of Proteins Related to Angiogenesis

**Figure 6:**
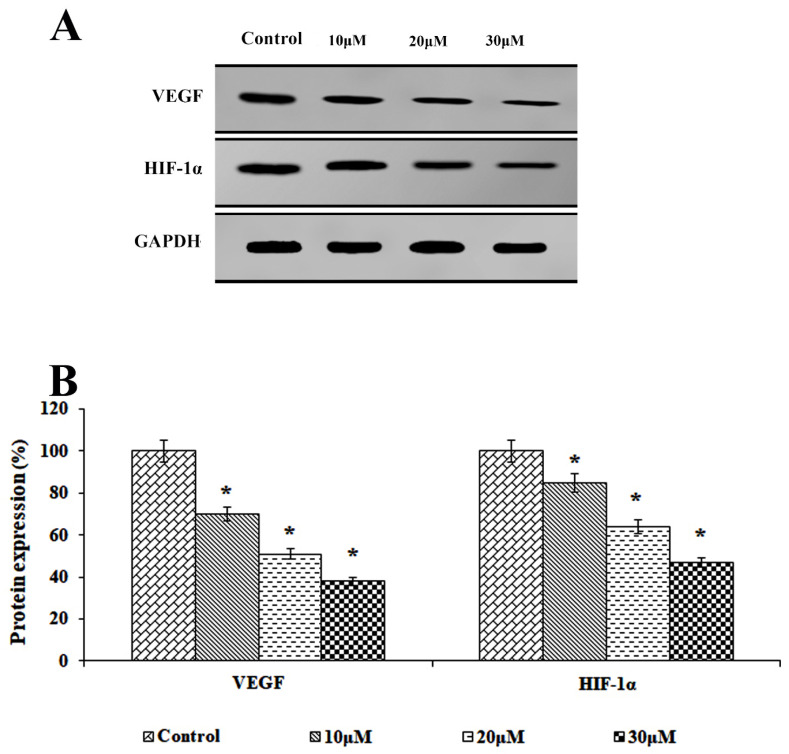
Effects of solanine on the expression of invasion-related proteins. (**A**) The expression of VEGF and HIF-1α in KB-ChR-8-5 cells after 24 h of treatment with and without solanine is shown in the representative immunoblot study. GAPDH served as a loading-control protein. (**B**) Densitometric analysis. Protein expression in the control lysates was assessed in triplicate and is represented as 100% in the graph. The statistical significance of the results was determined using an analysis of variance. The data are presented as the mean ± SD (*n* = 3). * *p* < 0.05 compared with the control.

This study aimed to elucidate the molecular pathways underlying the inhibitory effect of solanine on angiogenesis using Western blot analysis, as depicted in Figure 6. A concentration-dependent analysis conducted on KB-ChR-8-5 drug-resistant OSCC cells revealed that solanine has the capacity to downregulate the expressions of the proteins VEGF and HIF-1α at different concentrations (10, 20, and 30 μM). The results provide compelling evidence of solanine’s ability to suppress angiogenesis and the production of related proteins.

### 3.7. Effect of Solanine on Promote Apoptosis Protein Expression

**Figure 7:**
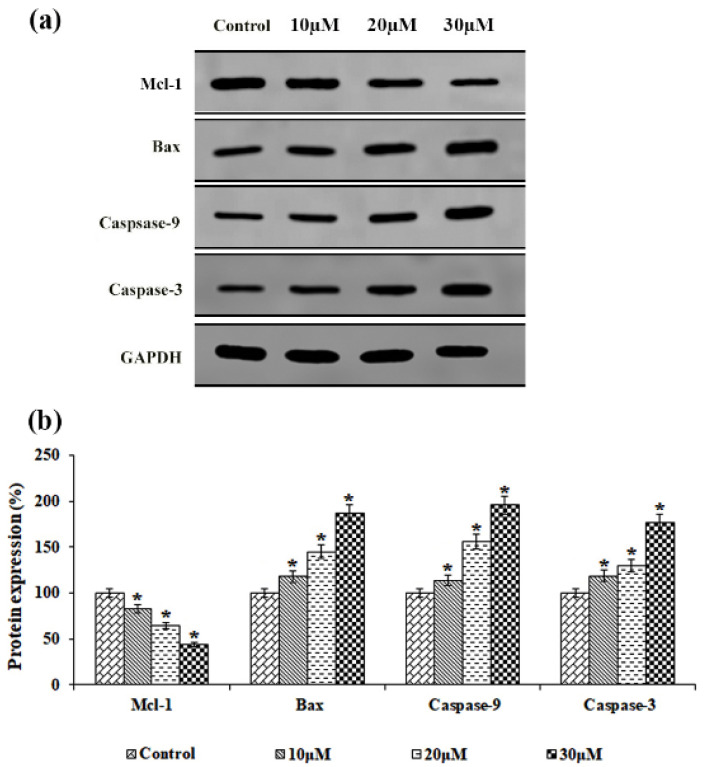
The effect of solanine on the expression of apoptotic proteins. (**a**) The expression of Mcl-1, Bax, Caspase-9, and Caspase-3 in KB-ChR-8-5 cells after 24 h of treatment with and without solanine is shown in the representative immunoblot study. GAPDH served as a loading control protein. (**b**) Densitometric analysis. Protein expression in the control lysates was assessed in triplicate and is represented as 100% in the graph. The statistical significance of the results was determined using an analysis of variance. The data are presented as the mean ± SD (*n* = 3). * *p* < 0.05 compared with the control.

The present study aimed to assess the expression of apoptotic markers in solanine-treated KB-ChR-8-5 multi-drug-resistant cancer cells, as shown in Figure 7. Notably, solanine treatment led to a significant decrease in Mcl-1 expression in cells. In contrast, cells treated with different doses of solanine (10, 20, and 30 μM) showed considerable upregulation of proteins that cause apoptosis, such as Bax, caspase-9, and caspase-3, which were expressed at significant levels. These results clearly imply a potential link between the activation of apoptosis and solanine therapy.

### 3.8. Effects of Solanine on EGFR-Mediated PI3K/AKT and Transcriptional Protein Signaling

**Figure 8:**
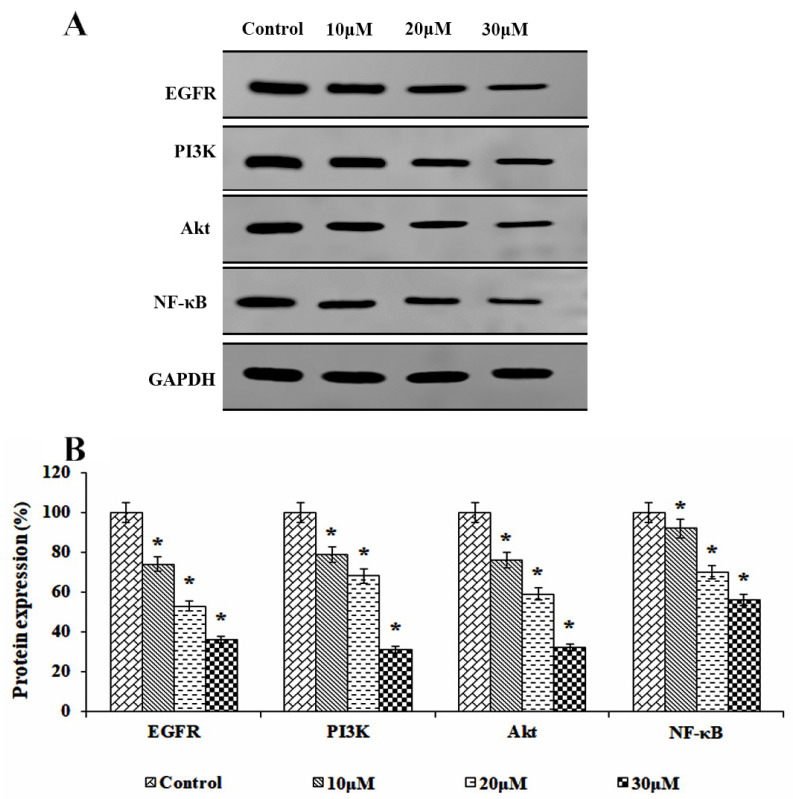
Effects of solanine on EGFR/PI3K/AKT/NF-κB protein expression. (**A**) The expression of EGFR, PI3K, Akt, and NF-κB in KB-ChR-8-5 cells after 24 h of treatment with and without solanine is shown in the representative immunoblot study. GAPDH served as a loading control protein. (**B**) Densitometric analysis. Protein expression in the control lysates was assessed in triplicate and is represented as 100% in the graph. The statistical significance of the results was determined using an analysis of variance. The data are presented as the mean ± SD (*n* = 3). * *p* < 0.05 compared with the control.

The study underscores the pivotal role of solanine in modulating EGFR-mediated PI3K/AKT/NF-κB signaling, which, in turn, regulates cell proliferation and angiogenesis in KB-ChR-8-5 drug-resistant OSCC cells. The application of solanine at varying concentrations (10, 20, and 30 μM) resulted in a significant reduction in EGFR expression and a marked decrease in the expression of PI3K and AKT, which further prevented nuclear translocation of NF-κB (Figure 8). These changes in protein expression contributed to the induction of apoptosis. The research underscores the substantial inhibitory effect of solanine on EGFR, which leads to the suppression of cell growth, angiogenesis, and the initiation of programmed cell death in drug-resistant OSCC cells.

## 4. Discussion

The activation of oncogenic receptors can lead to the uncontrolled growth of cells and the formation of angiogenesis that lead to drug resistance in cancer patients, resulting in the development of tumor progression. A new drug that targets and inhibits the function of cells responsible for angiogenesis, proliferation, and inflammation in tumors could extend cancer patients’ lives. The use of plant-based medicines in cancer therapy has gained popularity due to their potential to offer a therapeutic approach with minimal side effects. Solanine, a compound found in plants, has been shown to possess potent antioxidant and anti-cell-proliferation properties. Although different fundamental pharmacological investigations have demonstrated that solanine has anticancer effects [21], the extent of understanding limited solanine’s capacity to impede the growth of human carcinomas remains constrained, indicating a need for further investigation and comprehensive research. In our study, we observed that intervention with solanine affected the EGFR-mediated PI3K/Akt/NF-κB signaling pathway, effectively reducing inflammation, cell proliferation, and angiogenesis in aggressive drug-resistant oral carcinoma cells.

The development of drug resistance and the progression of oral squamous cell carcinoma (OSCC) can be influenced by high levels of active inflammatory factors, which can lead to DNA damage and genetic imbalance. Additionally, overexpression of proliferation markers has been linked to increased aggressiveness in angiogenesis and the prevalence of malignant tumors. Research has indicated that the activation of inducible nitric oxide synthase (iNOS) and interleukin-6 (IL-6) has the potential to elevate the risk of genetic modification and contribute to the advancement of chemoresistance and tumor progression [22,23]. Further, elevated levels of PCNA and cyclin-D1 have been associated with more aggressive behavior in oral squamous cell carcinoma (OSCC), leading to multidrug resistance and exerting a detrimental effect on the prognosis and overall outcome of patients [24,25]. Consequently, the inhibition of these proliferative and inflammatory proteins could be an attractive therapeutic approach for the treatment of OSCC. This study demonstrated that solanine exerted an inhibitory effect on the overexpression of iNOS, IL-6, PCNA, and cyclin-D1 in drug-resistant OSCC KB-ChR-8-5 cells, leading to the inhibition of inflammation and proliferation. These findings are consistent with previous reports on the anti-inflammatory and anti-proliferation effects of solanine in various other cancer types [26,27]. Thus, solanine could potentially be a key mediator of inflammation and proliferation, thereby inhibiting tumor growth in OSCC.

The dysregulated proliferation observed in OSCC cancer cells initiates a series of events, including the overabundant expression of angiogenic factors such as VEGF and HIF-1α. Consequently, this proliferation stimulates increased blood-vessel formation in healthy tissues. The development of malignancy is further extended by this complex process. This complex process exacerbates drug resistance and adversely affects patient survival [28]. Inhibiting the expression of VEGF and HIF-1α effectively suppresses angiogenesis and curbs tumor progression. Our study reveals that solanine successfully abrogates the expression of VEGF and HIF-1α in drug-resistant OSCC cells, which leads to the mitochondrial outer membrane permeabilizing, activating the mitochondrial pathway of apoptosis. Solanidine, a derivative of solanine, has shown itself to be effective in drastically attenuating the levels of HIF-1α and VEGF in human lung adenocarcinoma (A549) cells [29]. The results of this study contribute to a greater understanding of the mechanism of solanine’s anti-tumor activity, particularly in regard to the treatment and prevention of OSCC.

The elevated expression of proliferation and angiogenesis factors leads to a reduction in apoptosis in MDR cells exhibiting a malignant phenotype. Solanine is an antioxidant that suppresses MMP-induced Mcl-1 suppression and increases the expression of Bax, which promotes DNA strand breaks and caspase-mediated cell death. Thus, solanine, a naturally occurring substance, accelerates the activation of apoptosis by inhibiting proliferation and angiogenesis via the downregulation of transcription factors and upstream signals. Notably, it has been shown that the steroidal alkaloid solamargine, which has a structural similarity to solanine, may cause caspase-dependent apoptosis by regulating the expression of Bcl-2/Bax and may possess anti-carcinogenic properties in human renal carcinoma cells [30]. Nevertheless, the inhibitory effect of solanine on MDR cancer remains unclear.

The study illustrates that solanine treatment effectively impedes angiogenesis, suppresses cellular proliferation, and instigates apoptosis. However, further research is necessary to determine the specific pathways by which solanine inhibits drug-resistant oral squamous cell carcinoma. Recent studies on a range of drug-resistant cancers have consistently demonstrated that EGFR overexpression plays a pivotal role in driving both proliferation and angiogenesis [31,32]. The autophosphorylation of EGFR coordinates the simultaneous activation of PI3K/Akt signaling in cancer cells, facilitating the translocation of NF-κB to the nucleus. This translocation activates the transcription of target genes associated with proliferation and angiogenesis, ensuing genetic imbalance and the development of drug resistance [33,34,35]. The present study has revealed that solanine treatment effectively inhibits EGFR expression and exerts a downregulating influence on the PI3K/Akt/NF-κB activation cascade. As a result, this attenuation of signaling pathways leads to the suppression of inflammation, proliferation, and angiogenesis in drug-resistant cancer. Similarly, Lu et al. (2010) found that solanine significantly reduced the expression of the invasion proteins MMP-2 and MMP-9 by disrupting JNK-mediated PI3K/Akt signaling in human melanoma (A2058) cancer cells [36]. Gao et al. (2020) observed that in human liver cancer, solanine modulated the TGFβ/Smad signaling pathway to decrease tumour development [37]. Wen et al. (2016) demonstrated that solanine treatment suppresses vascular endothelial growth factor expression in human pancreatic cancer cells by down-regulating the ERK1/2-HIF-1α and STAT3 signaling pathways [38]. These outcomes underscore the anti-carcinogenic potential of solanine in multidrug-resistant cancer cells.

## 5. Conclusions

Solanine effectively reduces proliferation and angiogenesis in multidrug-resistant oral cancer cells (KB-ChR-8-5) by modulating the EGFR/PI3K/Akt/NF-κB signaling pathway. Treatment with solanine at concentrations of 10, 20, and 30 μM significantly increases the production of reactive oxygen species (ROS), leading to mitochondrial dysfunction and decreased mitochondrial membrane potential (MMP). This increase in ROS induces apoptosis, highlighting the role of oxidative stress in solanine-initiated cell death. Moreover, solanine significantly downregulates key inflammatory and proliferative markers, such as iNOS, IL-6, Cyclin D1, and PCNA, reducing tumor growth and inflammation. Additionally, the inhibition of the angiogenic factors VEGF and HIF-1α further supports the role of solanine in suppressing angiogenesis, which is critical for tumor progression. These findings collectively reinforce our conclusion that solanine acts as a potent anticancer agent by targeting multiple pathways involved in the proliferation and survival of drug-resistant cancer cells. Future studies will extend the investigation into the effects of solanine on the regulation of other tumor-suppressor pathways, both in vivo and in clinical studies. Therefore, solanine emerges as a promising candidate for the potential treatment of advanced oral cancer, serving as an effective anticancer agent.

## Data Availability

The data that support the findings of this study are available from the corresponding author upon reasonable request.
